# The Importance of *Acacia* Trees for Insectivorous Bats and Arthropods in the Arava Desert

**DOI:** 10.1371/journal.pone.0052999

**Published:** 2013-02-18

**Authors:** Talya D. Hackett, Carmi Korine, Marc W. Holderied

**Affiliations:** 1 Department of Biological Sciences, University of Bristol, Bristol, United Kingdom; 2 Mitrani Department of Desert Ecology, Swiss Institute for Dryland Environmental and Energy Research, Jacob Blaustein Institutes for Desert Research, Ben-Gurion University of the Negev, Midreshet Ben-Gurion, Israel; 3 The Dead Sea and the Arava Science Center, Tamar Regional Council, Neveh Zohar, Israel; Onderstepoort Veterinary Institute, South Africa

## Abstract

Anthropogenic habitat modification often has a profound negative impact on the flora and fauna of an ecosystem. In parts of the Middle East, ephemeral rivers (wadis) are characterised by stands of acacia trees. Green, flourishing assemblages of these trees are in decline in several countries, most likely due to human-induced water stress and habitat changes. We examined the importance of healthy acacia stands for bats and their arthropod prey in comparison to other natural and artificial habitats available in the Arava desert of Israel. We assessed bat activity and species richness through acoustic monitoring for entire nights and concurrently collected arthropods using light and pit traps. Dense green stands of acacia trees were the most important natural desert habitat for insectivorous bats. Irrigated gardens and parks in villages and fields of date palms had high arthropod levels but only village sites rivalled acacia trees in bat activity level. We confirmed up to 13 bat species around a single patch of acacia trees; one of the richest sites in any natural desert habitat in Israel. Some bat species utilised artificial sites; others were found almost exclusively in natural habitats. Two rare species (*Barbastella leucomelas* and *Nycteris thebaica*) were identified solely around acacia trees. We provide strong evidence that acacia trees are of unique importance to the community of insectivorous desert-dwelling bats, and that the health of the trees is crucial to their value as a foraging resource. Consequently, conservation efforts for acacia habitats, and in particular for the green more densely packed stands of trees, need to increase to protect this vital habitat for an entire community of protected bats.

## Introduction

Desert habitats are resource limited by definition, putting flora and fauna under particular constraints [1]. Anthropogenic disturbance of such extreme natural habitats can have long-lasting deleterious effects [2]. Within mammals, bats are the second most species rich order [3], provide valuable ecosystem services [4], are abundant in many habitats, can easily be monitored through recordings of their powerful sonar vocalisations and are good bioindicators of habitat quality [5]. In desert areas of Israel (e.g. Negev, Arava and Judean) there are 17 species of insectivorous bats, representing more than half of the country’s desert mammals [6], [7]. All insectivorous bats are protected by Israeli law and are either ‘vulnerable’, ‘near-threatened’ or ‘endangered’ on the International Union for Conservation of Nature (IUCN) red list for Israel [8].

Acacia trees are widely regarded as a keystone species with most desert fauna depending on them, either directly or indirectly, for food and shade [9]–[11]. They have an established positive impact on soil chemistry as nitrogen fixers [12] and increase herbaceous understory productivity [13]. Acacias hold crucial links to arthropods [14]–[16], such as ants, which live on acacias [17]–[20], bees which rely on acacia pollen [21] and bruchid beetles that infest seed pods [10], [17], [22], [23]. Gazelle (*Gazella dorcas*), Arabian oryx (*Oryx leucoryx*), small nocturnal omnivorous rodents (*Mastomys natalensis, Saccostomus campestris* and *Aethomys chrysophilus*) [24], [25], ostriches (*Struthio camelus*) and giraffes (*Giraffa camelopardalis*) [24] all consume the seeds of acacia, disperse and then fertilize pods aiding in germination while reducing the effect of seed parasites [23]. Three species, *Acacia tortilis, A. raddiana*, and *A. pachyceras*, provide the majority of wooded habitats in the Arava [26].

Acacia trees, particularly *A. raddiana* are in decline [27], [28]; the total mortality of acacia trees in the Arava Rift Valley may be as high as 61% over 14 years [9], [27]. This is primarily due to water stress, low recruitment of young acacia seedling and loss/change of habitat and water flow patterns [9], [27]. As acacia trees rely predominantly on surface water, the latter factor is of great concern [29]. Additionally, there is a significant decline in annual precipitation, which is likely to have a negative effect on mortality and recruitment of acacias [30]. Rohner and Ward [23] predict that loss of acacia trees in the Middle East would lead to a significant loss of biodiversity in the region.Despite the wealth of research on the ecology of acacia trees it is almost completely unknown how and to what degree bats and their nocturnal arthropod prey might utilize acacia trees. Vaughan and Vaughan [31] found that the central African bat *Lavia frons* uses *A. tortilis* and occasionally *A. elatior* as a night roost from which to forage, and suggest that the bats are feeding on insects that are attracted to acacia trees. In Australia *Vespadelus pumilus* selectively roost in *A. melanoxylon* despite their relative rarity in the area [32]. Moreover, surveys of bats in the Sinai [33], Kenya [34] and Swaziland [35] found bat foraging activity at sites that contained acacia trees. None of these papers examined a specific interaction between bats and acacia trees, nor was there any explicit comparison to other available foraging habitats.

Here we examine activity levels and species richness of insectivorous desert bats and the abundance and richness of their arthropod prey in available natural and artificial desert habitats, including irrigated agricultural sites (date palms) and villages where desert-dwelling species are attracted to artificial light sources [6], [36]. We hypothesise that acacias are a keystone genus in the nocturnal food web and therefore predict that nocturnal arthropods are diverse and abundant around acacia trees, and that bats are attracted to this foraging resource. Concurrently, we further hypothesise that the declining health of acacia habitats would negatively influence the bat and arthropod community and therefore predict that arthropod abundance and richness as well as bat activity and richness will be greater at dense green acacia stands than other available acacia habitats. Because artificial irrigation increases productivity in water limited ecosystems, we further predict that bat activity and arthropod abundance will be high in man-made habitats. We also predict that bat communities will differ between natural and man-made habitats, with a higher proportion of species that are typically recorded in the desert in natural habitats and more generalist synantropic species in the latter.

## Materials and Methods

### 2.1 Study Site

The Arava rift valley, which connects the Dead Sea to the Red Sea, is an extremely arid desert with approximately 25–50 mm of annual rainfall and an average summer temperature of 31°C [37]. The area is characterised by ephemeral rivers that flood briefly after occasional, often distant, rains in most winters but otherwise remain dry (wadis). Scattered small settlements with irrigated parks, gardens and agricultural fields exist along the entire length of the valley. Potential foraging habitats for insectivorous bats in the Arava thus range from open desert with scarce vegetation that is typically dry in the summer months, through wadis with shrubs or trees to artificially irrigated and lit settlements and agricultural fields (e.g. date palms). The most stable biomass producers in the desert resource web are trees belonging to the genus *Acacia*. They occur in scattered lines along some wadis and, less frequently, in dense assemblages which are typically near small, often seasonal, springs, that are drying up due to aquifer pumping and climatic changes [38].

### 2.2 Habitats

**Figure 1:**
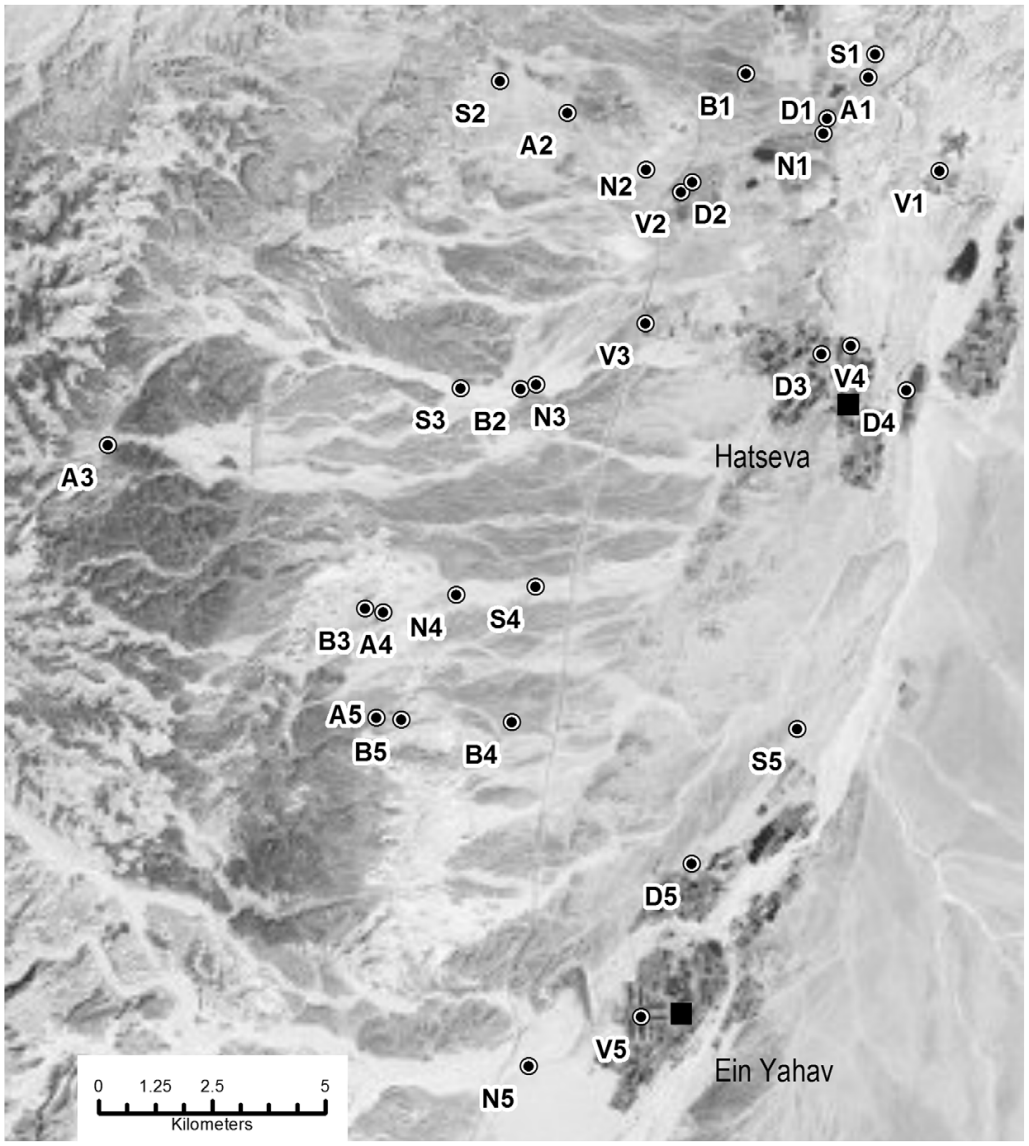
Satellite map of sites. A is dense acacia stands, S is sparse acacia stands, B is barren acacia stands, N is non-acacia desert sites, V is village sites and D is date plantations. The five replicates of each habitat type are numbered one to five from north to south (Reprinted with permission from Esri, original copyright 2012).

We studied six habitat types; four natural: (1) densely packed green acacia trees (predominantly *A. tortilis* and *A. raddiana*) with trees clustered less than 30 m apart, (2) sparsely distributed green acacia trees with trees separated by greater than 50 m, (3) brown/barren acacia trees and (4) desert sites without acacia trees, as well as two modified by human habitation: (5) agriculture in the form of date plantations, and (6) irrigated vegetation at walkways or gardens in villages. We selected five different locations for each of the six habitat types giving a total of 30 sites in a 20×15 km area between the villages of Idan and Ein Yahav and using, from north to south, the accessible wadis Bitaron, En Zach, Masor, Shehaq and Dohan. There were only five dense green acacia stands in the research area; one in each of the five wadis. All other natural sites were selected to span the same north-south range around these (numbered 1–5 from north to south) preferentially with one site of each habitat within each respective wadi. Because adequate habitats were not always available in the same wadi, one barren acacia (B1), one sparse acacia (S5) and one no acacia (N5) site had to be located outside of the five wadis, and two barren acacia sites (B4 and B5) were in one wadi. There were five settlements in the area so artificial sites were selected in and around each settlement again spanning the area in the north-south range (Fig. 1). No sites were within 100 m of water available for drinking and most were >500 m away. All natural sites were pristine and away from public illumination, and town sites were the only artificial habitats that had any non-natural lighting. Potential roosts in the form of caves and crevices were plentiful throughout the study region; buildings, occupied or abandoned, were located within likely bat commuting distance of all sites (<5 km). We collected data at the 30 sites from April to August 2009. We visited sites randomly but made sure to visit one site for all six habitat types before starting with the next set of six different sites. After all 30 sites had been sampled once, we repeated this two more times but in a different random order. To minimise any potential lunar effect, we did not sample for five days around the full moon.

### 2.3 Arthropod Sampling

We used a fluorescent light trap (Sylvania 15 W black light actinic bulb, 350 nm) suspended in front of a white cotton sheet to sample arthropods at each study site (for review of light trapping see [39]). Starting at 30 min after sunset, the light was turned on every hour for 30 minutes. All arthropods on the sheet were then collected during the following 10 minutes, and the light was then turned off for 20 minutes to avoid cumulative effects. We repeated this cycle four additional times and again once more beginning 90 min before sunrise. In villages, where ambient light might bias the attractiveness of our light trap, we placed the trap in darker areas, or those shielded from light. A pit trap was located less than 1 m from the sheet and checked every hour. We collected large arthropods in vials and smaller ones in pooters. This combination of methods has a bias against those arthropods not attracted to light but alternative methods were not suitable to the habitat/situation. Sweep netting was unviable as the net would get caught in acacia trees’ thorns damaging both the tree and the net while allowing all arthropods to escape; sticky traps quickly became covered in sand carried by the persistent winds; and, as many sites were in a national park, use of pesticides was not permitted. We identified all specimens at least to order and classified them as morphospecies. To create a reference collection of morphospecies hard-bodied specimens were collected, frozen and then pinned. Soft-bodied arthropods (Araneae, Scorpionidae and Solifugae) were stored in 70% ethanol. We analysed arthropod abundance per hour to account for periods of equipment failure in the light trap.

### 2.4 Acoustic Monitoring and Species Identification

**Figure 2:**
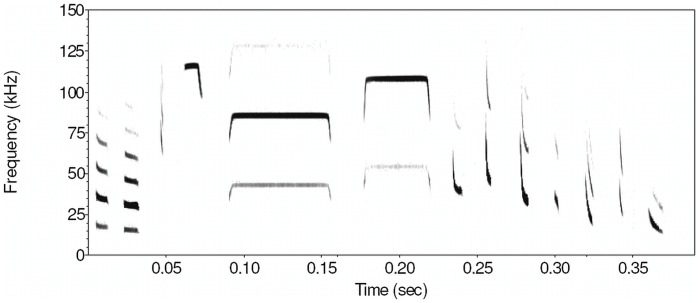
Spectrogram of one typical echolocation call from each identified species. From left to right: *Rhinopoma hardwickii, R. microphyllum, Nycteris thebaica, Asellia tridens, Rhinolophus hipposideros, R. clivosus, Pipistrellus kuhlii, Hypsugo bodenheimeri, Eptesicus bottae, Barbastella leucomelas, Otonycteris hemprichii, Plecotus christii* and *Tadarida teniotis*. Spectrogram parameters: FFT 1024, frame 100%, overlap 98.43%, window flat top.

At each site we used a full spectrum, direct recording automatic acoustic monitoring device with an omnidirectional microphone (BatCorder, EcoObs, Nuremberg, Germany) to record bat echolocation calls (@ 500 kHz and 16 bit) following the general approach outlined in Hayes et al. [40]. This device was hung from the edge of a tree 1–2 m from the ground. At sites where no trees were suitable a 1 m-high artificial stand was used. To avoid influencing recorded bat activity, the BatCorder was set at least 25 m from the arthropod trap. Once set, the BatCorder automatically records upon detection of a bat call and continues recording as long as bat calls are detected. After 800 ms of silence it ceases recording until triggered by a new call which starts a new file. Detection was assumed to be equal in all sites since desert habitats are all acoustically transmissible, with little canopy cover even at dense acacia sites. Within villages, sites were also open and there were no tall buildings obstructing bat flight. Agricultural sites were the most densely covered, but trees were still spaced 8–10 meters apart with crown diameters that leave gaps of 2–4 m between trees. There is currently no quantitative data on the transmissibility of habitat types for ultrasound, but large gaps in foliage in all habitats and the omnidirectional microphone of the BatCorder [41] mean that this is unlikely to have been a strong effect in this region. Due to differences in the source levels of different species’ echolocation calls, “loud” aerial hawkers are likely to be recorded over greater distances than “whispering” gleaning species [41]; this bias could not be eliminated but was equal across all sites. Activity was measured as number of bat passes per night and each recording file was defined as one pass. This is a conservative measure of bat activity if two passes of the same species are separated by less than 800 ms of silence. Multiple species present in the same file were defined as separate passes [42]. In a pilot survey in summer 2008, we identified the bat species foraging around acacia trees in the Arava using a combination of recordings from hand-released bats and descriptions of echolocation calls from studies in the broader region [33], [43]. We established that desert bats in Israel can be identified to species level based on species/specific echolocation call design (see Fig. 2).

We used a weather monitoring device (Silva ADC Pro, Silva Sweden AB, Sweden) to record temperature, humidity and wind speed at the position of the BatCorder, with measurements taken one hour after sunset. At the beginning the night, wind was often blowing constantly with speeds of 5–10 km/h and occasionally as high as 20 km/hr. At a variable time, typically before midnight, this wind stopped abruptly and conditions remained calm for the rest of the night. Because of this pattern, wind was recorded as either present or absent one hour after sunset coinciding with the usual peak foraging activity. To standardise bat species identification and efficiently process the large number of recordings, we developed an automatic classification algorithm in SasLab Pro v. 4.40 (Avisoft Bioacoustics, Berlin, Germany). Peak frequencies at the start, end and maximum amplitude were measured for each echolocation call, which was then classified to species using defined frequency ranges per species. When compared to manual species identification the automatic classification correctly identified 95% of bat passes across all species (695 passes over 3 nights from pilot data in 2008); errors occurred when the recorded calls were too faint for the automatic classification to pick up (13 out of 115 passes for *Rhinopoma hardwickii,* 7 out of 537 passes for *Hypsugo bodenheimeri*), and when a *H. bodenheimeri* call overlapped with a *R. hardwickii* call it was mistakenly classified as *Eptesicus bottae* (5 passes); the passes of all other species were correctly identified in all cases. In order to reduce any further errors, some files had to be checked manually for potential misclassifications. This was necessary for recordings where (a) no call was classified/detected, or where (b) all calls classified as *Otonycteris hemprichii, Plecotus christii* or *Tadarida teniotis*, because low frequency noise was sometimes mistakenly classified one of these species. Echolocation calls from *O. hemprichii* and *P. christii* differ characteristically in the end frequencies, duration and the amount of spectral overlap between the first and second harmonic, but they had to be separated manually because automatic classification was unreliable. There were also rare misclassifications between solitary calls of three species with peak frequencies of approximately 30 kHz (*E. bottae, R. hardwickii,* and *R. microphyllum*). Hence, all files containing two or fewer such calls were also checked manually. Mist nets were routinely placed at each site, with the intention of confirming activity estimates, but due to the open nature of the desert habitats we rarely caught bats, stressing the advantages of acoustic monitoring for bat surveys. Bat captures and surveys were conducted under license #34615 given to CK by the Israel Nature and Park Authority, and all sites were visited with permission from land owners or the Israel Nature and Park Authority.

### 2.5 Statistical Analysis

Bat passes, arthropod abundance and the total number of bat species recorded at each site were heteroscedastic, therefore a log_10_(x+1) transformation was applied to the data enabling the use of parametric tests. A mixed-model analysis of variance (ANOVA) with one between-subjects factor (habitat) and one within-subjects factor (visit number) was performed on bat passes and arthropod abundance/hour. Green, dense acacia habitats were then individually compared to all other habitats using pairwise t-tests with sequential Bonferroni adjustment. Bat and arthropod species richness was measured as the number of species or morphospecies present at each site during the period of study. An ANOVA was performed on the number of bat species and arthropod morphospecies across habitats. A Pearson correlation test was performed to determine the relationship between bat passes and arthropod abundance. To test for any confounding effect of abiotic factors (temperature, humidity and wind) we performed a multivariate ANOVA for each visit cycle on the total number of bat passes per night and arthropod abundance per hour per night; we used a Bonferroni correction to control for multiple tests. All statistics were computed and graphs created using R-2.7.1 statistical environment (The R Foundation for Statistical Computing, 2008).

## Results

We collected a total of 46,471 arthropods almost exclusively at the light trap over 533 hours with only 10 arthropods in pit traps. We identified 733 arthropod morphospecies in the following systematic groups: Lepidoptera (234), Coleoptera (100), Orthoptera (31), Mantoidea (12), Diptera (109), Neuroptera (30), Hymenoptera (39), Hemiptera (144), Blattaria (5), Odonata (4), Dermaptera (5), Isopoda (2), Ixodida (1), Pseudoscorpionida (1), Aranae (13), Solifugae (1) and Scorpiones (2).

**Figure 3:**
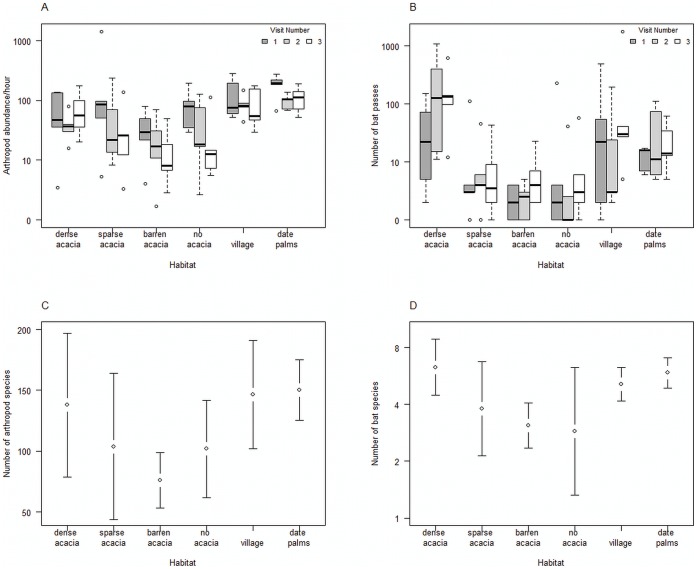
Arthropod abundance, bat activity and species richness for each habitat. **a**: box plot of arthropod abundance per hour for three consecutive repeats (visits). **b**: box plot of total number of bat passes for three consecutive repeats (visits). **c**: total number of arthropod morphospecies (mean ± standard deviation. **d**: total number of bat species recorded (mean ± standard deviation).

Arthropod abundance was affected by both habitat type (F_5,24_ = 3.94; p  = 0.009; fig. 3a) and visit number (F_2,48_ = 5.26, p = 0.009; Fig. 3a). Arthropod abundance was lower at dense green acacia trees than at date sites (t_21_ = 3.42; p = 0.012), but after correcting for multiple testing there was no difference compared to other habitats (sparse acacia trees: t_24_ = 0.29, p = 0.77; barren acacia trees t_27_ = 2.55, p = 0.067; no acacia trees: t_26_ = 0.96, p = 0.69, village sites t_24_ = −2.19, p = 0.11). The number of arthropod morphospecies did not change significantly between habitat (F_5,24_ = 2.27, p = 0.079; Fig. 3c).

We identified 13 bat species by their echolocation calls (Fig. 2): *Rhinopoma hardwickii, R. microphyllum, Nycteris thebaica, Asellia tridens, Rhinolophus hipposideros, R. clivosus, Pipistrellus kuhlii, Hypsugo bodenheimeri, Eptesicus bottae, Barbastella leucomelas, Otonycteris hemprichii, Plecotus christii* and *Tadarida teniotis*. We caught seven of these species in mist nets: *R. hardwickii, A. tridens, R. clivosus, H. bodenheimeri, E. bottae, O. hemprichii* and *P. christii.*

Over a total duration of 963 hours on 72 recording nights we recorded 6,575 bat passes in 5,586 files. Typically each file contained a single pass by a single individual, but passes of different species sometimes occurred in the same file indicating that multiple species were foraging at the same place and time. The number of bat passes was affected by both habitat type (F_5,24_ = 3.55; p  = 0.015; Fig. 3b) and visit number (F_2,48_ = 3.84, p = 0.028; Fig. 3b). The number of bat passes was significantly greater in dense green acacia trees than in all other natural desert habitats (sparse acacia trees t_27_ = 4.05, p = 0.001; barren acacia trees t_20_ = 5.88, p<0.001; no acacia trees: t_28_ = 4.23, p = 0.001) and date fields (t_22_ = 2.43, p = 0.05); but was not significantly different from village sites (t_27_ = 1.47, p = 0.15).

**Figure 4:**
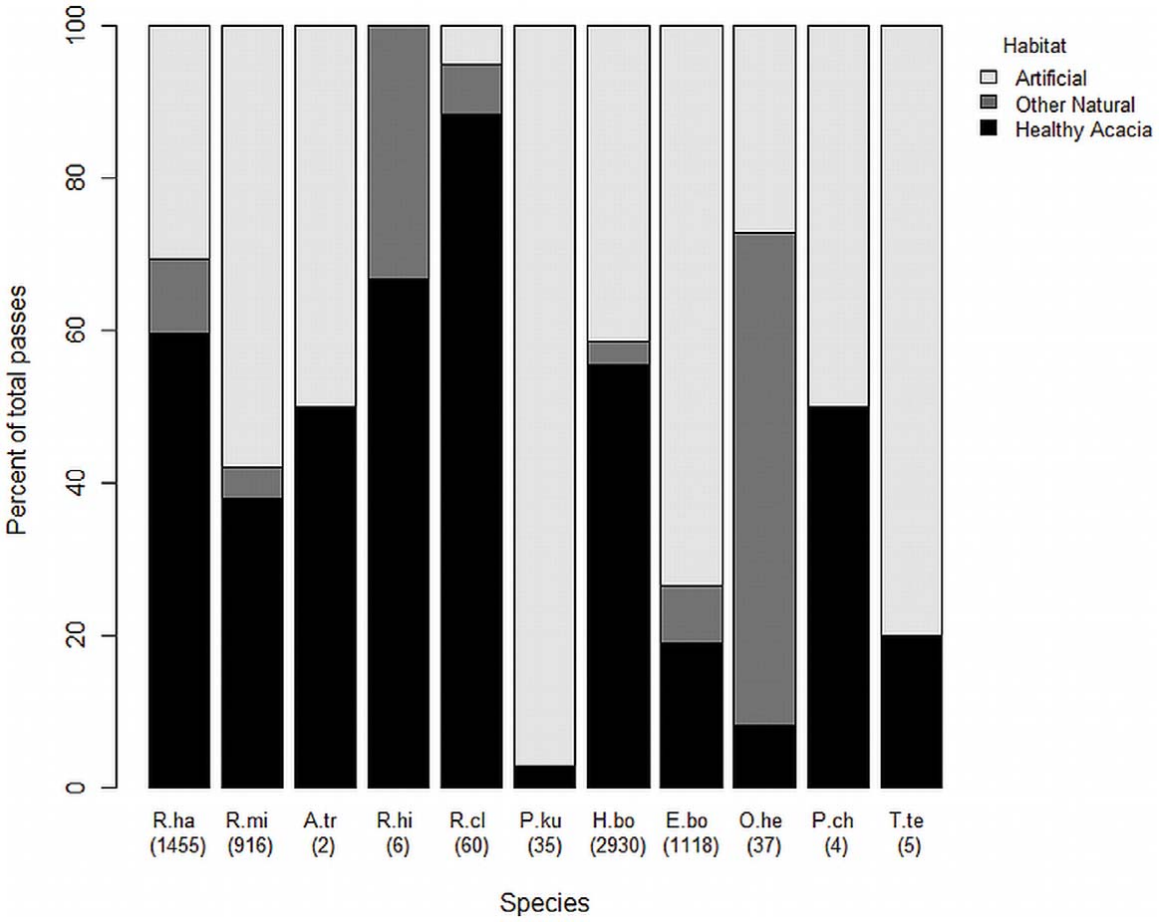
The percentage of recorded bat passes in each habitat per species. R.ha: *Rhinopoma hardwickii*, R.mi: *R. microphyllum*, A.tr: *Asellia tridens*, R.hi: *Rhinolophus hipposideros*, R.cl: *R. clivosus*, P.ku: *Pipistrellus kuhlii*, H.bo: *Hypsugo bodenheimeri*, E.bo: *Eptesicus bottae*, O.he: *Otonycteris hemprichii*, P.ch: *Plecotus christii*, T.te: *Tadarida teniotis*. Numbers in brackets indicate the total number of passes for that species.

The number of bat species present was affected by habitat type (F_5,24_ = 2.80, p = 0.042; Fig. 3d), with dense green acacia trees having more species than barren acacia trees (t_8_ = 3.50, p = 0.04), but not significantly different from any other habitat (sparse acacia trees: t_8_ = 1.64, p = 0.44; no acacia trees: t_8_ = 5.54, p = 0.36; village sites: t_8_ = 6.40, p = 0.60 and date palms: t_8_ = 6.25, p = 0.75). Some species of bat were recorded almost exclusively in desert habitats and, within them, mostly in healthy acacia stands while others were more likely to be recorded in non-natural habitats (Fig. 4). For instance Rhinolophid species were recorded almost exclusively in natural habitats while *P. kuhlii* and *T. teniotis* were almost exclusively found in artificial sites. Other species (e.g. *R. hardwickii* and *H. bodenheimeri*) were more equally distributed between habitats.

**Figure 5:**
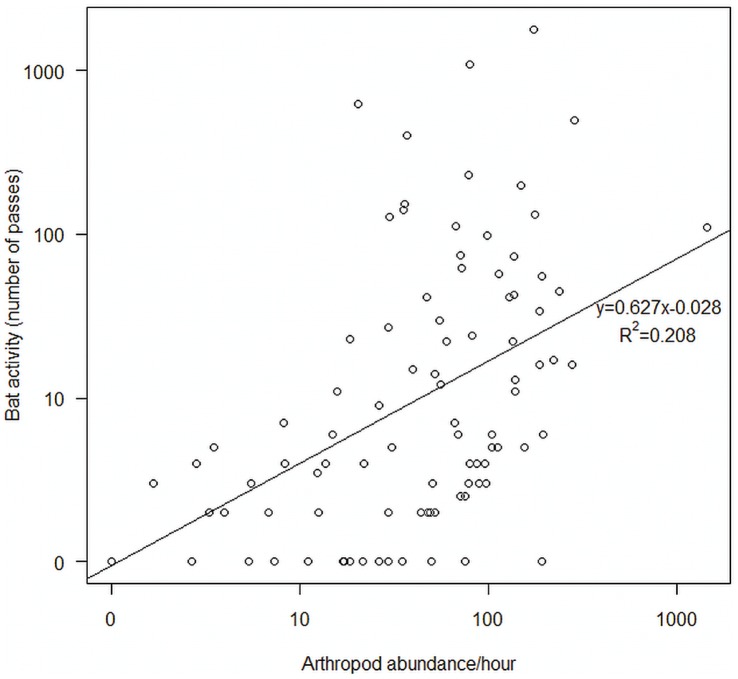
The relationship between arthropod abundance and bat activity. Each data point represents one entire night of sampling. Solid line is a linear regression (y = 0.627×−0.028; R^2^ = 0.21; t_88_ = 4.81, p<0.001).

There was a positive correlation between the number of bat passes and the arthropod abundance across all habitats but only 20.8% of the variation is accounted for by this relationship (R^2^ = 0.21; t_88_ = 4.81, p<0.001; Fig. 5). Wind had an effect on arthropod abundance in each visit cycle (1^st^ visit: F_1,28_ = 12.73, p = 0.011; 2^nd^ visit: F_1,28_ = 13.81, p = 0.008; 3^rd^ visit: F_1,28_ = 9.20, p = 0.048) but not on bat activity (all visits F_1,28_<1.92, p>0.18). No other abiotic factor affected either arthropod abundance or bat activity (all F_1,28_<8.20, p>0.07).

## Discussion

In accordance with our hypothesis, insectivorous bat activity was higher in dense green acacia stands than any other natural habitat, and species richness was high at habitats with dense green acacia trees. While dense green acacia trees only differed significantly from barren acacia trees in terms of bat species richness it was only at green acacia sites that we recorded all 13 species, which make up 76% of insectivorous bat species known from the deserts of Israel and Jordan [33], [44], [45]. Since natural sites were located along wadis, they may all be used as commuting routes. Thus the difference in bat activity levels, but not in species richness, between dense green acacias and the other natural desert habitats could be a result of bats flying through sparse acacia and no acacia sites en route to dense green acacias. These results indicate that healthy stands of acacia trees are key natural foraging resources for desert-dwelling insectivorous bats.

There was however only a weak link between habitat type and arthropods; no natural habitat differed in arthropod abundance or richness from green, dense acacias. One possible explanation for this is that our arthropod trapping method is biased towards light-attracted species, thus we are likely under sampling arthropods not attracted to light in all habitats. There might be a bias if these species’ abundance differed between habitats. While we attempted other sampling methods, these were not effective or not viable. The effect of visit number on both the number of bat passes and abundance of arthropods indicates that there is a potential seasonal component to habitat profitability and use. Healthy acacias remain green all year but partition flowering seasonally [46], and were in full flower during the 3^rd^ visit. There is a general trend for arthropod abundance to decrease across the visits, particularly the 3^rd^ visit, in the more barren sites (barren acacia trees and natural non-acacia sites). However, at green acacia trees (both dense and sparse) the level remains high during the 3^rd^ visit in midsummer. It is therefore likely that green flowering acacias become even more important for bats and nocturnal arthropods as summer progresses and other habitats get less productive.

As we predicted, artificially irrigated and lit man-made habitats did have high arthropod abundance and bat activity. Date palms supported a greater abundance of arthropods than dense green acacias, while village sites and dense green acacias had equally high levels of bat activity. Moreover, arthropod and bat species richness for both date palms and village sites did not differ significantly from dense green acacias. These findings support the observation that for some species of bat, man-made habitats can, in fact, act as an alternative foraging resource [6].

Our results are consistent with previous studies that found bat activity correlated with arthropod abundance [47], [48]. We recorded a range of bat species with different dietary niches [49], thus the activity of some species would likely correlate better with specific species of arthropods than others. Further studies into the diet of the bats in this area are needed to clarify this.

As predicted, some bat species relied more heavily on acacia trees than others; all species recorded here are listed at least as regionally vulnerable [8] (Table 1). Use of green acacia habitats was strongest in species typically recorded in deserts: *R. clivosus*, which mainly catches flying Coleoptera near vegetation, where echoes may come from objects that are not the target (cluttered environment) [6], [44], [49]; and *O. hemprichii*, which gleans terrestrial arthropods from surfaces [49]–[52] and tends to forage in xeric, sparsely vegetated, rocky environments [53] that are usually cluttered [6]. Conversely, *P. kuhlii*, a generalist in terms of prey and habitat selection that favours habitats with street lights [49], [54], and *T. teniotis,* which hunts for flying insects in open spaces but is a generalist in terms of prey selection [6], [33], [49], [54] were encountered mainly in non-natural habitats. Four species were recorded approximately equally in all habitats, both natural and artificial. All of these are aerial insectivores foraging in background cluttered space: *H. bodenheimeri*, is known to be a generalist in terms of both habitat and prey selection [6], [49], [55], [56]; *R. hardwickii* forages on aerial Coleoptera and swarming Hymenoptera in open habitats [49], [57]; *R. microphyllum* predominantly consumes Coleoptera and is often found in sympatry with *R. hardwickii* [57], [58]; and *E. bottae*, is a background cluttered space aerial insectivore [6]. Thus, artificial habitats created by the settlements appear to at least partially compensate for the habitat loss, but only for some of the desert species. Moreover, *P. kuhlii*, a species which has only expanded its range into desert areas of Israel following human habitation, could compete for resources with desert specialists in the vicinity of settlements [36], [44], [59], [60].

**Table 1:** Regional and global conservation status of recorded bat species.

Species	Regional status [8]	Global status [83]
*Rhinopoma hardwickii*	Vulnerable	Least concern
*Rhinopoma microphyllum*	Vulnerable	Least concern
*Nycteris thebaica*	Endangered	Least concern
*Asellia tridens*	Vulnerable	Least concern
*Rhinolophus hipposideros*	Vulnerable	Least concern
*Rhinolophus clivosus*	Vulnerable	Least concern
*Pipistrellus kuhlii*	Near threatened	Least concern
*Hypsugo bodenheimeri*	Endangered	Data deficient
*Eptesicus bottae*	Vulnerable	Least concern
*Barbastella leucomelas*	Endangered	Least concern
*Otonycteris hemprichii*	Vulnerable	Least concern
*Plecotus christii*	Endangered	Least concern
*Tadarida teniotis*	Near threatened	Least concern

Three species were rarely recorded (Fig. 4): *A. tridens* and *R. hipposideros* tend to forage in highly cluttered environments [6], [49], [61]–[63], and *P. christii* is a recently isolated whispering bat presumed to consume mainly Lepidoptera [64]. *A. tridens* and *P. christii* were sampled equally at healthy acacia and artificial sites while *R. hipposideros* was recorded mostly at healthy acacia sites and never in artificial habitats. Artificial light has been shown to negatively influence activity levels of *R. hipposideros* so it would not be expected in, or very near to, villages [65].

Of particular interest are two additional rare species that were only recorded outside our analysed sampling period, but exclusively at dense green acacia sites: *B. leucomelas* and *N. thebaica*. *B. leucomelas* has been caught only five times before in Israel. We have recorded it five times at three different dense green acacia tree sites. As they are so rare, nothing is known about habitat selection of *B. leucomelas* and this is the first occurrence of consistent recordings in the region. *N. thebaica* is a generalist/opportunistic feeder [49], [66] found foraging in open savannah woodland areas [66]. It is also a whispering bat, hunting in flight or from a perch [66], [67]. As these two as well as *P. christii* and *O. hemprichii* are presumed whispering bats with low intensity calls, they will have been under sampled and in fact be more prevalent in the area than determined by acoustic monitoring [68].

Many studies have found increased bat activity at sites with water [6], [69], [70], but that is not likely to be the explanation for the site-dependant differences we recorded. Sites of different habitats were all an approximately equal flight distance from standing water. Moreover, during the summer months the natural pools and springs often dry up completely, yet activity levels remained high.

Environmental factors are suggested to play a role in where bats forage. Temperature is negatively correlated with activity levels [71]–[76], while heavy rains can stop all foraging [71], [72], [77], and relative humidity is positively correlated to bat activity [78]. Environmental conditions remained similar during the period of each cycle of 30 sites, with a temperature range of less than ±5°C and without any precipitation. We found no significant effect of either temperature or humidity on either arthropod abundance or bat activity. The abiotic factor most likely to influence bat activity in the Arava is strong wind, because this increases energy expenditure for powered flight [79]. The effect of wind on bats is somewhat ambiguous, with evidence of both no change in bat activity [71] as well as a decrease in activity [54], [78]. We did not find an effect of the presence of wind on bat activity over the whole night, but did on arthropod abundance, possibly because the wind moved the collection sheet thereby preventing insects from landing. Since wind was discontinuous and stopped abruptly during the night it is possible that bats shifted their activity to calm periods; thus leaving bat activity levels over the entire night unaffected.

Kunz et al. [4] reviewed the ecosystem services provided by bats, concluding that insectivorous bats potentially exercise a top down control of arthropods in both natural and agricultural ecosystems. The use of exclusion nets to determine the relative effects of predation by birds and bats on arthropods indicates that there is an equal or stronger effect of bats on arthropod abundance [80]–[82]. Moreover, bat predation of arthropods had an indirect effect on herbivory, providing a strong case for bats as biological agents of pest control [81], [82]. Thus, the high activity level and diversity of insectivorous bats we found around dense healthy assemblages of acacia trees and also in irrigated agriculture might indicate that bats act as a biological control agent in both natural and agricultural habitats in the Arava.

Our findings provide evidence that acacia habitats are keystone foraging sites especially for rare bat species and desert specialists. Irrigated habitats in deserts are frequented by a selection of desert species, but synantropic species might increase resource competition [60]. We also give evidence that the health of the tree has a strong influence on activity level. Acacia trees’ further decline will have a significant impact on bats that forage in these areas. There is a need for better conservation and protection, particularly given the protected status of insectivorous bats in Israel and the ecosystem services they can provide.
